# A simpler and more cost-effective peptide biosynthetic method using the truncated GST as carrier for epitope mapping

**DOI:** 10.1371/journal.pone.0186097

**Published:** 2017-10-12

**Authors:** Wan-Xiang Xu, Jian Wang, Hai-Ping Tang, Ling-Han Chen, Wen-Bo Lian, Jian-Min Zhan, Satish K. Gupta, Chao-Neng Ji, Shao-Hua Gu, Yi Xie

**Affiliations:** 1 Division of Reproductive Immunology, Key Lab of Reproduction Regulation of NPFPC, Shanghai Institute of Planned Parenthood Research, Fudan University, Shanghai, P. R. China; 2 Reproductive Cell Biology Laboratory, National Institute of Immunology, Aruna Asaf Ali Marg, New Delhi, India; 3 State Key Laboratory of Genetic Engineering, Institute of Genetics, School of Life Science, Fudan University, Shanghai, P. R. China; Shanghai Medical College, Fudan University, CHINA

## Abstract

There is a need to develop better methods for epitope mapping and/or identification of antibody-recognizing motifs. Here, we describe improved biosynthetic peptide (BSP) method using a newly developed plasmid pXXGST-3 as vector, which has a viral E7 gene in the cloning sites of pXXGST-1. It is crucial to employ pXXGST-3 instead of pXXGST-1, since it makes use of the BSP method simpler and easier to perform, and more cost-effective for epitope mapping. These merits are embodied in two aspects: i) convenient recovery of double enzyme-digested product due to the existence of 315 bp inserted between *BamH* I and *Sal* I sites, and thus greatly reducing the production of self-ligation clones, and ii) no longer requiring control protein when screening recombinant (r-) clones expressing 8/18mer peptides by running polyacrylamide gel electrophoresis. The protocol involves the following core steps: (i) design of plus and minus strands of DNA fragments encoding overlapping 8/18mer peptides; (ii) chemical synthesis of the designed DNA fragments; (iii) development of r-clones using pXXGST-3 vector expressing each 8/18mer peptide fused with truncated GST188 protein; (iv) screening r-clones by running the cell pellets from each induced clone on SDS-PAGE gel followed by sequencing of inserted DNA fragments for each verified r-clone; and (v) Western blotting with either monoclonal antibodies or polyclonal antibodies. This improved GST188-BSP method provides a powerful alternative tool for epitope mapping.

## Introduction

For rational vaccine design or development of diagnostics, it is imperative to map all functionally relevant, specific and/or conserved linear (continuous) B cell epitopes (BCE) of target proteins. Several methods have been described to define linear BCEs, which can be broadly classified into four categories: i) chemically synthetic peptide (CSP) and its chip method [1–5]; ii) biosynthetic peptide (BSP) by gene fragment expression, phage display random-peptide or antigen-fragment libraries and expression-PCR technique, etc [6–11]; iii) epitope prediction based on computational tools relating to several physical or chemical parameters of a given protein; and iv) sequencing of proteolytic fragments applying enzymatic and chemical reagents. However, performance of these methods, including their numerous improved or derivative methods, is far from ideal because of the various limitations. In particular, for classical CSP method commonly used, it is not easy to identify antibody-recognizing BCE minimal motifs even when using monoclonal antibodies (mAbs) in most cases, or impossible to reveal IgG-epitome on a target antigen using polyclonal antibodies (pAbs) that is the basis of finding all desired functional BCEs.

**Fig 1:**
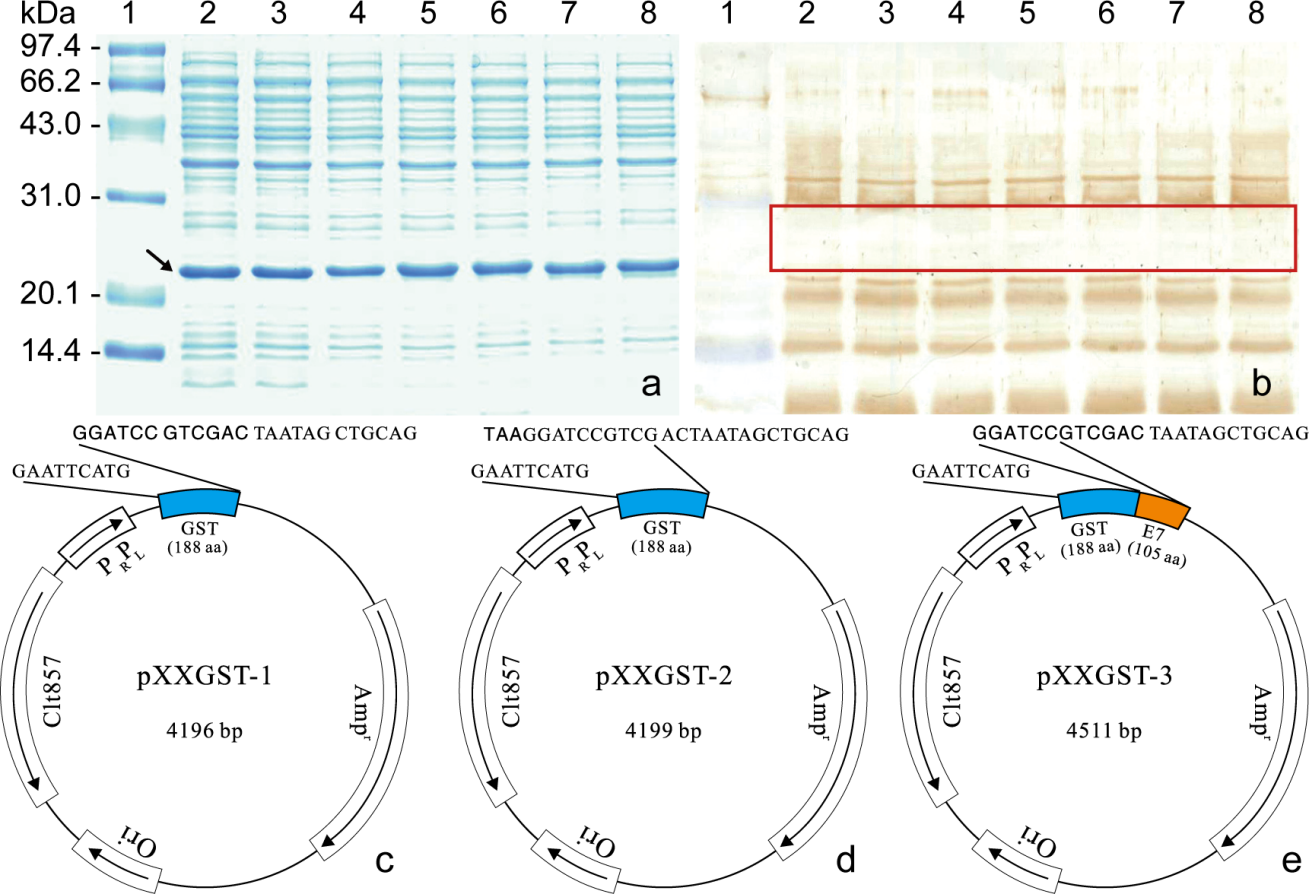
Weak antigenic range of cell proteins in Western blot and schematic diagram of relevant plasmids. (a) SDS-PAGE analysis of expressed GST170-8mer peptides and (b) Western blotting with rabbit antiserum generated against the r-segment b (aa residues 177–348) of human zona pellucida glycoprotein-3 (huZP3b), and (c-e) Schematic diagram of pXXGST-1, pXXGST-2 and pXXGST-3. The arrow indicates the expressed various 8mer peptides fused with GST170 protein (subpanel a), and the red box shows a maximum weak antigenic area (subpanel b). The boldfaced letters in cloning site and other colors in pXXGST-1 and -3 indicate enzyme restriction sites of *BamH* I and *Sal* I, and gene fragments encoding GST188 and GST188-E7 proteins, respectively.

Recently, many research groups tried to use BSP method similar to CSP method for antibody identification or BCE mapping of antigenic segments of interest, on which several genes encoding core streptavidin (Stv), glutathione S-transferase (GST), and β-galactosidase (Gal) were used as carriers of BSP [12–17]. These explorations, including our construction of minimum short fragment encoding 4 amino acids (aa) fused with Stv gene [12] and finding of weak antigenic range (~21 to 30 kDa) in bacterial proteins (Fig 1A and 1B), resulted finally in the development of original BSP method capable of arbitrarily expressing overlapping 8/18mer peptides. It employed a truncated GST gene encoding 188 residues (GST188) as carrier in pXXGST-1 (Fig 1C) [18], and the subsequent improvement of applying the enhanced chemiluminescence (ECL) reagents in Western blotting for BCE mapping [19].

Although the method has been used to reveal IgG-epitomes of three major proteins from human papillomavirus type 58 (HPV58) and in other related studies [18–25], it still had potential faults that needed to be overcome for convenience of users, which were: i) it is unable to purify the double enzyme-digested pXXGST-1 product with 4190 base pairs (bp), because it is only 6 bp shorter than the 4196 bp product generated by inadequate digestion leading to lower efficiency of constructing recombinant (r-) clones; and ii) setting GST188 control was required to screen the r-clone of expressing GST196 (GST188 + 8mer peptide) protein due to the ~ 1 kDa of migration rate difference between both proteins, otherwise it is inconvenient to distinguish the ~ 0.5 kDa difference between GST196 and GST192 (GST188 + 4 aa) proteins expressed by r- and self-ligation clones. It also has same trouble for determining self-ligation clone by running SDS-15% polyacrylamide gel electrophoresis (PAGE), due to the ~ 0.5 kDa difference between the GST192 and control GST188 proteins that were expressed by pXXGST-1 and pXXGST-2 (Fig 1D). It is likely that these clones expressing GST192 protein could be mistakenly regarded as r-clones and subsequently followed-up for sequencing leading to wasteful expenditure.

In our newly improved method (named GST188-BSP), these shortcomings have been well addressed through using pXXGST-3 (Fig 1E), which can make it more convenient and efficient for cloning and screening r-clones of overlapping 8/18mer peptides, because of the strategy to insert a longer DNA fragment between both target cloning sites of pXXGST-1. Obviously, the 315 bp in length of selected E7 gene is sufficient to distinguish two different enzyme-cut products of 4190 bp and 4511 bp, and the ~ 11.5 kDa of E7 protein composed of 105 aa can make the GST188-E7 fusion protein out of the 21 to 30 kDa weak antigenic area, and thus no longer require any protein control for screening r-clones of 6/18mer peptide due to non-existence of any nonspecific band like GST192 protein within the area of cell proteins from a self-ligation clone of pXXGST-3. The GST188-BSP method presented here not only keeps characteristics and virtues of original BSP approach but also is simpler and easier to perform, and more cost-effective. Here, we describe the method and its standardized protocol for general laboratory application. Our data suggest that the GST188-BSP method reported herein is an excellent alternative tool for BCE/epitome mapping and identification of mAb-recognizing minimal motif.

## Materials and methods

### Ethics statement

The preparation of rabbit antisera to r-huZP4C_227-463_ segment was conducted in accordance with the Guidelines for the Care and Use of Laboratory Animals, and was approved by the Ethics Committee of Shanghai Institute of Planned Parenthood Research (SIPPR). The procedures were in accordance with guidelines established by the Ethics Committee of SIPPR. Ethical approval for the unpublished research project No. 2012BAI31B07 was granted by the ethical committee of the SIPPR (2012–08).

### Immunization of animals

Three male New Zealand white rabbits purchased from SIPPR-BK Lab Animal Co., Ltd. (Shanghai, China) were caged at controlled temperature of 25°C. All the performed experiments were in full compliance with standard laboratory animal care protocols approved by the Institutional Animal Care Committee of SIPPR. These rabbits were immunized intramuscularly with 1 mg of purified *E*. *coli*-expressed recombinant human zona pellucida glycoprotein-4 corresponding to amino acid residues from 227 to 463 (r-huZP4C_227-463_) emulsified in 0.5 ml of complete Freund’s adjuvant and 0.5 ml of PBS at multiple sites on rabbit’s back. Three booster immunizations of 0.5 mg r-huZP4C_227-463_ protein in incomplete Freund’s adjuvant were administered at 3-week intervals. Sera were collected from each rabbit seven days after each vaccination and stored at -70°C.

### Plasmids and antibodies

The thermo-inducible plasmids of pXXGST-1 and pXXGST-2 were constructed as described earlier [18]. The plasmid pXXGST-3 was constructed by our group, which had a HPV18-E7 gene (GenBank No: X05015) inserted between *BamH* I and *Sal* I sites in pXXGST-1, but failed to express GST188-E7 fusion protein in the various prokaryotic expression systems used by us. The rabbit antisera raised against r-huZP3b_177-348_ [26] and r-huZP4C_227-463_ segments were prepared previously, the mAb C1P5 [27] (American Research Products Inc., USA), and goat anti-rabbit/mouse IgG conjugated to horseradish peroxidase (HRP) (Proteintech Group, USA) were purchased from Shanghai Sangon Co., China.

### Other reagents and materials

DNA ligase, restriction enzymes *BamH* I and *Sal* I were purchased from Takara Co., Ltd (Dalian, China). *Escherichia coli* (*E*. *coli*) strain BL21 (DE3) (Novagen, Inc., USA) competent cells, QIAprep Spin Miniprep Kit and QIAquick Gel Extraction Kit (QIAGEN, Duesseldorf, Germany), prestained molecular weight markers (Shanghai Shisheng Cell Biotechnol, China), 0.2 μm nitrocellulose membrane (Whatman GmbH, Germany), enhanced chemiluminescence (ECL) plus Western blotting detection kit, and other general chemicals were obtained from Shanghai Sangon Co., Ltd, China.

### DNA fragment synthesis and sequencing

All gene fragments encoding short peptides of interest were synthesized by the SBS Genetech Co., Ltd, Shanghai. DNA sequencing of inserts in each r-clone was performed by Shanghai Generay Biotechnology Co., Ltd, China.

### Molecular cloning

The molecular cloning of synthesized DNA fragments was done as described previously [28], which has been briefly described in the ‘Protocol’ as described later in this section.

### Protocol of BSP method for characterization of BCE

#### Step one: Designing series of overlapping short peptides

Design all 16/18mer peptides with an overlap of 8 aa residues covering the full-length of the target protein or a longer segment sequence for the first round of antigenic peptide mapping, as well as design a set of 8mer peptides with overlapping 7 aa residues and covering each reactive 16/18mer peptide sequence for the second round of precise BCE motif identification.

Note: It is recommended to design overlapping 16mer peptides, as the cost for synthesizing oligonucleotide of less than 60 nt in length is lower than that of oligonucleotide of more than 60 nt. Also, when using a mAb or cross-reactive mAb to map BCE, it is desirable to express two or several longer segments of the target protein so as to determine its shorter antibody reactive segment followed by designing overlapping 8/16mer peptides. Doing so can save the cost, time and workload in following steps 4–7.

#### Step two: Designing oligonucleotides of plus and minus strands

**Fig 2:**
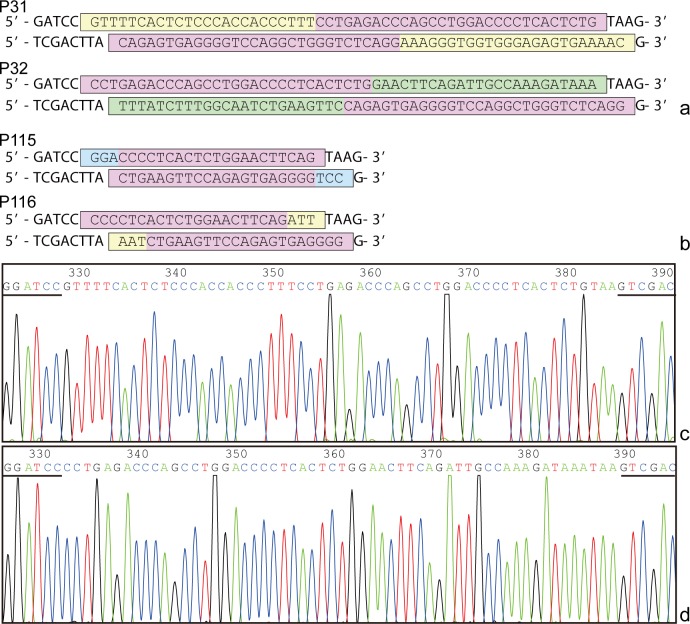
Design of inserting DNA fragments encoding short peptides and DNA sequencing. (a, b) BSP-based design for both plus and minus strands of huZP4 gene fragments encoding 18/8mer peptides, and (c) sequencing result of plus strand inserted fragment encoding P31. Pink color in all boxes indicates the paired bp in each double strand and overlapping regions of 10/7 aa between designed P31-32 and P115-116. Other colors indicate various paired bp in four of double strands. The underlines in Fig 2c indicate *BamH* I and *Sal* I restriction sites.

Note: It is emphasized that design of the target gene fragments at this step is very convenient, that is, the consideration of the optimal codon usage for expression of each short peptide fused with GST188 in *E*. *coli* is not essential as more than 1000 of the constructed 8/18mer peptide fusion proteins have been successfully expressed by using their DNA fragments from known prokaryotic and eukaryotic genes [12–14, 19–25]. However, it should be ascertained that whether there is another potential *BamH* I or *Sal* I site within the oligonucleotide sequence, if any, it needs to be modified because it will disturb annealing of the paired oligonucleotides and its subsequent cloning.

Design all plus and minus strands of DNA fragments encoding each designed 8/18mer peptides of the target protein, having cohesive ends for *BamH* I and *Sal* I restriction enzymes at their 5’and 3’ ends, so that they could be directly used to insert into the *BamH* I and *Sal* I sites downstream of the GST188 gene in plasmid pXXGST-3 after their annealing in pairs. Also, a TAA termination codon is designed between each 8/18mer peptide and *Sal* I site, so as to avoid possible false blotted bands of 8/18mer peptides for BCE and minimal motif mapping, which will be caused by four GSVD residues encoded by 12 bp present in cloning site region of pXXGST-3 (Fig 1E). In short, the designed plus and minus strands of encoding fragment should include sequences of 5’- GATCC and TAAG-3’, as well as 5’- TCGACTTA and G-3’ at their both ends (Fig 2A and 2B). As mentioned in step one, it is just close to 60 nt that is oligonucleotide corresponding to 57 nt encoding 16mer peptide together with 9 nt at their both ends.

#### Step three: Chemical synthesis of the designed DNA fragments

Once the plus and minus stands of encoding gene fragments have been designed, they can be synthesized and PAGE-purified by the SBS Genetech Co., Ltd, Shanghai. All PAGE-purified DNA fragments should be stored at -20°C prior to use.

#### Step four: Conduct annealing reactions of PAGE-purified DNA fragments in pairs

1. To make 100 μM stock solution of each synthesized DNA fragment by adding appropriate volume of double distilled H_2_O (ddH_2_O) into tubes based on the report provided by the biotechnical service company, respectively.
2. Take respectively 5 μl of the stock solution from two tubes in pairs into a 1.5 ml of new tube, and add 90 μl of ddH_2_O to make 5 μM final concentration of each DNA fragment.
3. Heat tubes up to 94°C in an electric heating block, anneal for 5 min, and then allowed them to cool gradually to room temperature (RT).

#### Step five: Development of r-clones for expressing each 8/18mer peptide

1. Perform the ligation reactions using above each annealed DNA fragment and constructed vector of pXXGST-3 digested with *BamH* I and *Sal* I enzymes, and then purified using QIAquick Gel Extraction Kit after running 0.7% agarose gel electrophoresis at 50 V for 90 min. The ligation reaction was carried out at 16°C overnight using the following 15 μl of reaction mixture in 0.5 ml tube (Table 1), in which all components were mixed by flicking and spin briefly (refrigerated Mikro 120 Centrifuge).
2. Employ the above ligation mixture in each tube to transform the *E*. *coli* BL21 (DE3) competent cells according to the standard procedures [28]. Grow overnight each clone on solid Luria Broth (LB)-ampicillin (amp) plates at 37°C, respectively. It should be observed that there are about 10 to 20 clones on the LB plates next morning.
  Note: If there was no growth of clones on a LB-amp plate after repeating twice, it suggests that they were incorrect or wrongly paired DNA fragments and need to be resynthesized.
3. Grow overnight each clone from LB-amp plates in 3 ml of liquid LB-amp medium at 30°C, subsequently dilute 1:50 in fresh LB-amp medium and were grown at 30°C at 220 rpm to an OD_600_ of 0.6–0.7. The clones were induced at 42°C for 4–5 h. Harvest each cell pellets by centrifugation at 5000 x g for 5 min at 4°C. Cell pellets obtained from 3 ml culture of expressed 8/18mer peptide fusion proteins were boiled in 400 μl of 1x sample loading buffer (50 mM Tris-HCl, pH 6.8, 2% SDS, 0.1% bromophenol blue, 10% glycerol and 100 mM β-mercaptoethanol) for 5 min.

**Table 1:** Component of ligation reaction.

Component	Amount
10 x T4 DNA ligase buffer	1.5 μl
DNA fragment (0.05 μmol μl^-1^)	2 μl
pXXGST-3 vector (0.01 μmol μl^-1^)	1 μl
T4 DNA ligase (350 U μl^-1^)	1.2 μl
ddH_2_O	up to 15 μl
Total volume	15 μl

#### Step six: Screening of r-clones by running SDS-PAGE

Take ~3 μl of each cell suspension from prepared individual thermo-induced bacterial cultures to run SDS-15% polyacrylamide gel for screening r-clones, and cell proteins resolved by conducting electrophoreses at 100 V for 2 h. The gels were stained with Coomassie Brilliant blue G250 for analyzing the bands of fusion proteins.

**Fig 3:**
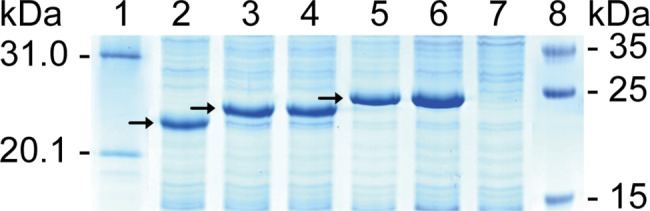
SDS-PAGE analysis of expressed 8/18mer peptide fusion proteins for screening r-clones. Lane 1/8, prestained protein markers; lane 2, GST188 protein expressed by pXXGST-2; lanes 3–4, 8mer peptides P115-116 of huZP4; lanes 5–6, 18mer peptides P31-32 of huZP4; and lane 7, negative control for self-ligation of pXXGST-3. Arrows show the control GST188 or each 8/18mer peptide fusion proteins.

Note: As shown in Fig 3, all r-clones can be easily determined by observing difference between GST188 fusion proteins expressed in lanes 3–6 and control GST188 protein expressed by pXXGST-2 in lane 2. Approximately 1 kDa difference can be observed even when expressing overlapping 8mer peptide fusion proteins as compared to GST188 protein. However, as shown in lane 7, there was no specific 8/18mer-peptide fusion protein band appeared as compared to lanes 3–6 when self-ligation of pXXGST-3 happened due to inadequate enzyme digestion, suggesting that it no longer needs any additional control (lane 2 or 7) on the gels for the 8/18mer peptides expressed specifically in r-clones when using pXXGST-3 vector in the improved GST188-BSP method.

#### Step seven: Sequencing of insert for each determined r-clone

Conduct sequencing of all synthesized DNA fragments inserted into plasmid pXXGST-3 from r-clones verified by above SDS-PAGE analysis through the Shanghai Biosune Biotechnology Co., Ltd, China. It is recommended to use the primer 5’-GGCCATCATACGTTATATAG-3’.

Note: For ensuring that all amino acid sequences of expressed short peptides are accurate, it is important to check the correctness of each synthesized DNA fragments by sequencing after determining r-clones and before conducting BCE and minimal motif mapping (Fig 2C), because this step can avoid possible error in BCE mapping due to possible bp missense mutation or tubes numbering errors happened when synthesizing a large number of DNA fragments. Therefore, elimination of deleterious errors becomes necessary in many cases. Of course, for saving cost and time if needed, it might be carried out after finishing two rounds of BCE and fine motif mapping to check those DNA fragments encoding reactive 8/18mer peptides.

#### Step eight: Determination of BCE recognized by mAbs/pAbs in Western blot

Proteins on SDS-PAGE gel were electrotransferred onto 0.2 μm nitrocellulose membrane, on which complete transfer was ensured by staining membrane with 0.1% Ponceau S. Non-specific antibody-binding sites on the membrane were blocked with blocking phosphate-buffered saline (PBS) containing 0.05% Tween 20 and 1% skim milk powder or preimmune rabbit serum sample overnight at 4°C, and probed with antisera raised against r-huZP4C that was pre-adsorbed with host cell lysates or mAb C1P5 (1:1000 or 1:5000 dilution in PBS) for 2 h at RT. Following washing four times, specific bands on the membrane were visualized by using goat anti-rabbit IgG or goat anti-mouse IgG conjugated with HRP at 1:10000 dilution. The blot was developed by using the ECL plus Western blotting detection reagents.

Note: To produce clear blotted bands in Western blotting, it is suggested that before use, the antiserum against r-protein may be absorbed on host cell lysates as described earlier [28], which facilitate in removal of cross-reactive antibodies to *E*. *coli* proteins.

## Results

### Fine BCE-motif mapping with pAbs

**Fig 4:**
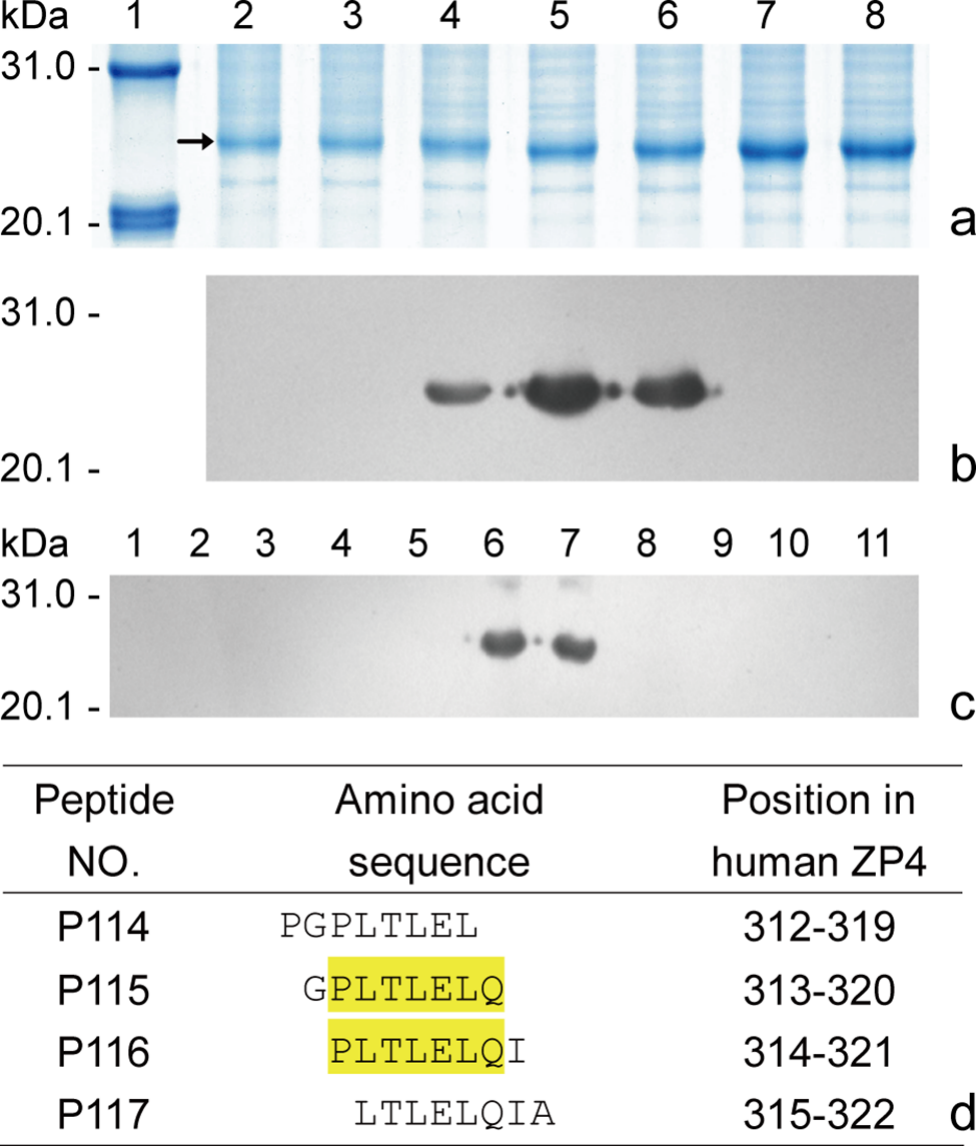
Fine BCE-motif mapping of reactive P32. (a) SDS-PAGE analysis of expressed 18mer peptides (lane 1, prestained protein markers; P30-36 in lanes 2–8) fused with GST188; (b, c) Western blotting of 18mer and 8mer peptides using rabbit sera to r-huZP4C (8mer P110-120 in lanes 1–11); and (d) the precise motif analysis. The yellow highlight in reactive P115-116 peptides indicates the shared sequence recognized by antisera to r-huZP4C. The arrows indicate the 18mer peptides of expressed P30-36.

In order to know how many BCEs are there among three neighboring reactive 18mer-peptides of P32-34 (Fig 4A and 4B), which were blotted in first round of antigenic peptide mapping in our study on epitome decoding of huZP4 protein, we first expressed a set of 8mer-peptides (P110-120) covering the full-length sequence of reactive P32 according to procedures of steps 1 to 8 in the protocol, and then they were subjected to SDS-PAGE, and transferred onto 0.2 μm nitrocellulose membrane. The Western blotting was performed by standard method with 1:2000-diluted rabbit antisera to r-huZP4C. Specific antigen–antibody reactions on the membrane were visualized by applying goat anti-rabbit IgG conjugated to HRP and ECL plus Western blotting detection kit with higher sensitivity according to the manufacturer’s instructions.

As shown in Fig 4C and 4D, a precise BCE motif (PLTLELQ) was defined in reactive P32 according to the common sequence highlighted in yellow color within reactive P115-116 peptides. The mapped motif was in good agreement with the PLTLEL motif of huZP4 that was recognized by rabbit anti-native porcine ZP IgG in another previous study [18], which only had a residue Q at its C-terminus more than that of the latter, suggesting that rabbit immune system could recognize the common antigenic site shared among homologous proteins of pig and human, no matter whether they were against native protein or not.

**Fig 5:**
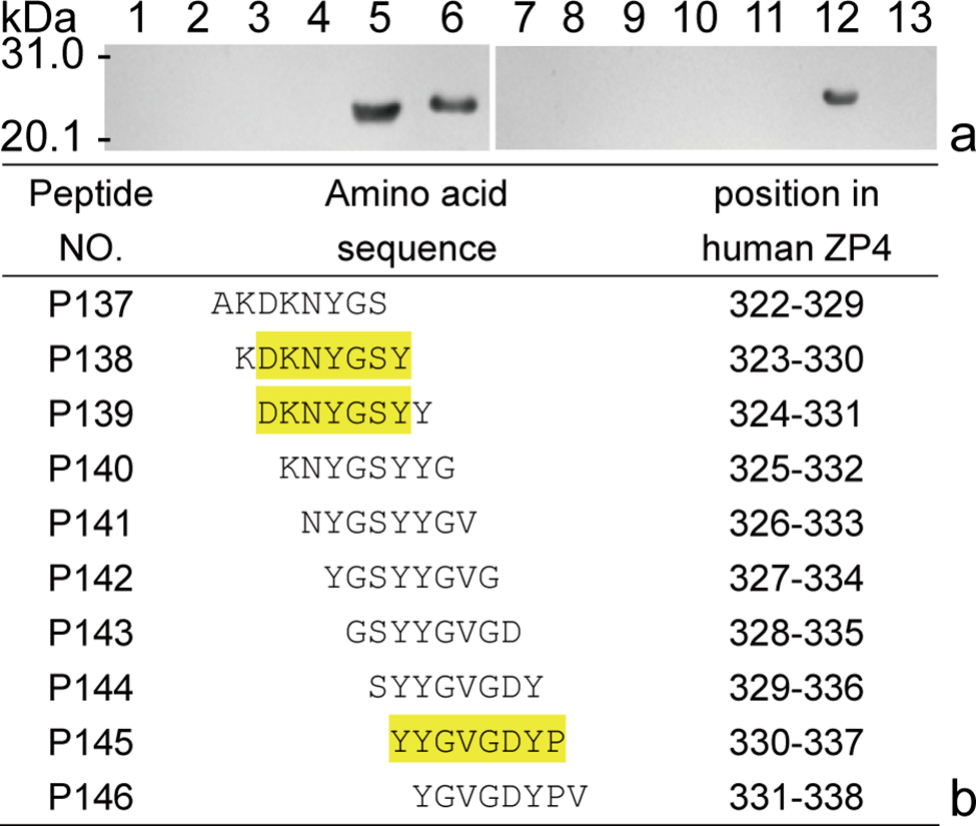
Fine BCE-motif mapping of reactive P33-34. (a) Western blotting of 8mer peptides using rabbit sera to r-huZP4C (8mer P135-146 in lanes 2–13); and (b) the precise motif analysis. The yellow highlight in reactive P138-139 and P145 peptides indicates the shared sequence and single reactive 8mer peptide recognized by antisera to r-huZP4C.

Obviously, it is impossible to identify a BCE among these reactive peptides (P32-34) using rabbit antisera to r-huZP4C, if not applying the GST188-BSP method. By the way, other two BCE motifs in reactive P33 and P34 have also been identified using a set of overlapping 8mer peptide of P34 in completed study on epitome decoding of huZP4 glycoprotein, which one (DKNYGSY^324-330^) was present in the 10 aa overlapping region of P33-P34 and another (YYGVGDYP^330-337^) at the C-terminus of P34 (Fig 5).

### Fine BCE motif mapping with a mAb

There is a need to identify the fine BCE motif recognized by a non-conformational mAb, and the present GST188-BSP method is a powerful and economic technique for such purpose. To save cost and workload, express two or more bigger segments of a large protein at first so as to determine a reactive segment, and then map BCE motif using 18/8mer peptides for a specific mAb. For a known cross-reactive mAb; however, another strategy may be adopted to find one or several potential segments of 100% conservation or with a residue difference among homologous proteins through sequence alignment. The following is a practical example for the latter case.

**Fig 6:**
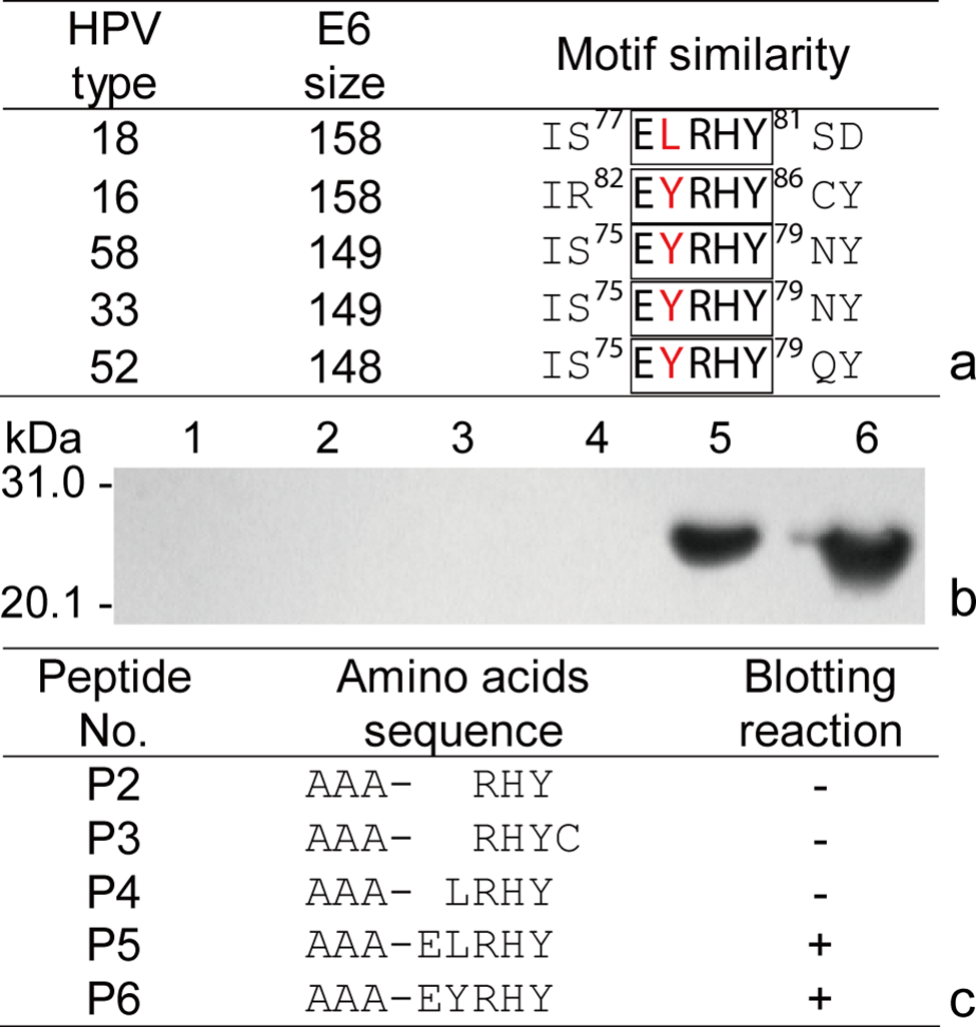
Fine BCE-motif mapping of mAb C1P5 from HPV18-E6. (a) Sequence alignment of E6 proteins from HR-HPVs; (b) Western blotting with mAb C1P5 (P2-6; lane 1, GST188 negative control); and (c) Design of five short peptides. The + and–signs in (c) indicate the reactivity of mAb C1P5 with P2–P6 fusion proteins.

The fine BCE E6-2 motif (YGD/XTL) of HPV58-E6 was found to be highly conserved among most known high risk-HPVs (HR-HPVs) including HPV16 and HPV18 in our previous study [20], and the commercially available mAb C1P5 of HPV18-E6 can cross-react with HPV16-E6 protein [27]. Therefore, in order to know whether the mAb also could recognize the E6-2 epitope, the Western blotting tests were performed using our previously expressed P35 octapeptide containing YGDTL motif and r-E6 protein of HPV58. The mAb did not react with P35, but recognized r-protein of HPV58-E6. Thus, another highly conserved site (EL/YRHY) with only a residue difference among them was found via sequence alignment of E6 proteins from HPV16, 18 and 58 (Fig 6A).

Based on BCE minimum motif of three aa [19, 20], thus, a panel of DNA inserts encoding 3mer to 5mer peptides (the addition of three AAA residues at their N-terminus is only for the convenience of constructing r-clones) were designed, synthesized, annealed, and cloned into *BamH* I and *Sal* I sites within pXXGST-3, resulting in five r-vectors. After constructing and expressing each r-clone, cell pellets from induced r-clones were used to perform Western blotting with mAb C1P5. Finally, the BCE fine motif (ELRHY) of HPV18-E6 and a cross-reactive pentapeptide (EYRHY) among several homologous proteins were characterized, which the consensus pentamer sequence EL/YRHY was based on blotted results of P5 and P6 (Fig 6B and 6C). Meanwhile, it was yet found from the mapping result that besides HPV16-E6, the cross-reactive mAb C1P5 can recognize HPV58-E6 as well as oncogenic HPV33- and 52-E6 proteins that were according to subsequent EYRHY alignment of homologous E6 proteins (Fig 6A), suggesting the importance of identifying fine BCE motif for a cross-reactive non-conformational mAb.

### Cost analysis of biosynthesis of 8/16mer peptides

In Shanghai, People’s Republic of China, the cost of oligonucleotide synthesis is currently about USD 0.17/nt (RMB yuan 1.20/nt) for oligonucleotides of length less than 60 nt (57 nt for encoding 16mer peptide that included 9 nt of cohesive sequence at their both ends), and USD 0.29 (RMB yuan 2.00) per base for length more than 60 nt (63 nt for encoding 18mer peptide). The DNA sequencing cost of insert for every r-clone is about USD 2.85 (RMB yuan 20). For converting the cost of DNA fragment into peptide synthesis, it needs USD 1.40/aa for 16mer peptide [RMB yuan 9.80/aa (57 x 2 x 1.20 + 20) / 16], and USD 1.77/aa for 8mer peptide [RMB yuan 12.40 (33 x 2 x 1.20 + 20) / 8]. The rough calculated average total cost for producing a 8mer or 16mer BSP was about USD 1.71/aa (RMB yuan 12.00/aa) that included costs of use of enzymes (DNA ligase, *BamH I* and *Sal* I), molecular weight markers and other reagents until got a sequence-confirmed r-clone, which is far below present market price (USD ~15.7 to 19.40 or RMB yuan ~110.00 to 135.00 per residue in Shanghai Sangon Co., China and the Biomatik Co., USA) of 6/18mer CSP with >98% purity used in BCE mapping. The cost-effectiveness of the GST188-BSP method is obvious when conducting BCE fine motif or epitome mapping to a macromolecular protein. Most importantly, it can obtain more definitive and trustworthy results of BCE mapping by applying such BSPs than CSPs [20], and these r-plasmids expressing 8/18mer BSPs can also be stored at -20°C for future possible use.

## Discussion

Most of the methods pertaining to mapping of BCEs have employed CSP strategy [29], but it failed to reveal epitome of a target antigen using this strategy due to the limitation of being unable to use pAbs to map a BCE fine motif and/or more within several contiguous reactive peptides as shown in Fig 4 that resulted in a limited number of mapped BCEs [30–32]. For example, not including a BCE present in the C-terminus transmembrane-like domain, only three BCEs on mature huZP3 were identified by rabbit pAbs in ELISA assay when using a panel of overlapping 8mer CSPs covering huZP3 sequence [30], whereas it was shown by our group that there are at least seven BCEs present in the protein by Western blotting with rabbit pAbs and sets of 8mer BSPs [19].

For non-conformational mAbs, there are several reports on using sets of overlapping 8/10mer CSPs or 12mer peptide library to identify BCE minimal motifs [33–35]. Due to limited availability of 8/10mer peptides synthesized on polypropylene pins and the high cost to purchase CSPs of high-purity, this method has limited applications. Further, most of mapped 16mer antigenic peptides were inadvertently referred as “epitopes” [14–17], even being 37mer peptide [36], although it is well recognized that smaller number of the aa residues are needed for binding to the antibody [37]. It is likely that within a mapped 18/20mer peptide there may be two and more BCEs [19, 20, 38–40]. Moreover, the mAb-recognizing fine motif has not yet been stipulated as a needful criterion or parameter for manufacturing a mAb(s) as drug, although all know it is one of the major features to distinguish one mAb from another, and could broaden application range of a mAb. For instance, the commercially available mAb C1P5 raised against HPV18-E6 protein can be used to identify HPV33/52/58-E6 as well, besides known HPV16-E6 according to the mapping results in Fig 6.

In early explorations of BSP, the Stv118 (expression plasmid pTSA18), GST245 (pGEX-6p) and Gal590 (pWR590) proteins were used as carriers to express 4/16mer peptides for BCE mapping or antibody identification [12–17]. These endeavors suggested the feasibility to develop BSP approach in addition to CSP strategy. However, these efforts failed to show the merits of BSP approach leading to a standard protocol suitable for general laboratory application. Major drawbacks of applied BSPs in above studies were: i) expressed 16mer peptide fusion proteins need to be purified before Western blotting even when using chicken sera to SARS-CoV and SARS convalescent sera [14–16], with this operation step it obviously increases the research cost and work load, and takes much longer time; ii) it is inconvenient to select each r-clone of 16mer BSPs before sequencing of insert, which needs to conduct the double enzyme restriction to identify insert, and iii) the position of expressed short peptides fused with GST245 or Stv118 was not in the widest weak antigenic range of bacterial proteins, and thus was unsuitable for BCE mapping with pAbs, etc.

The present GST188-BSP method as described has fully embodied practical approach. The outstanding advantages of GST188-BSP method are as follows: i) using the truncated GST188 as carrier, it makes the expressed 8/18mer peptides fusion proteins in the weak antigenic area of bacterial proteins, and thus permits directly using them to map each BCE fine motif and epitome of target protein; ii) employing pXXGST-3 enables users to extract double enzyme-cut pXXGST-3 by running agarose gel electrophoresis, and thus greatly reduce the self-ligation of pXXGST-3 via removing products produced by inadequate digestion that may happened sometimes; iii) screening r-clones on the SDS-PAGE gels no longer needs any control, because as shown in lane 7 of Fig 3, there are no band similar to GST192 protein in the 21 to 30 kDa area of cell proteins, which will interfere with screening r-clones of 8mer peptide in the 21 ~ 30 kDa area for self-ligation of pXXGST-3; iv) employing thermo-inducible expression system instead of isopropyl-β-D -thiogalactoside (IPTG) induction reduces cost; v) it is less expensive than CSP method due to cheaper cost of oligonuleotide synthesis than peptides; vi) it is clearer and more credible for the blotted bands in Western blotting due to use of the ECL luminescence reagents than others such as 3,3’-diaminobenzidine (DAB) or alkaline phosphatase (AP) kit; vii) it is adaptable for most laboratories due to its relative simplicity.

In conclusion, based on the improved GST188-BSP method using the pXXGST-3 vector, we have constructed more than one hundred of r-clones to express 8/18mer and minimum 3mer peptides (via adding three alanine residues to its N-terminus, Fig 6C) fusion proteins in our ongoing studies of epitome mapping. The epitopes recognized by pAbs to r-huZP4C and mAb CIP5 against HPV18-E6 have been mapped. Hence, we believe that the GST188-BSP method offers a much simpler option which is more cost-effective, reliable and adaptable for general laboratories. It will facilitate studies on epitope/epitome mapping of numerous target proteins and BCE motif identification of non-conformational mAbs.
